# Limitations in representative sampling of unpaid caregivers from minority ethnocultural backgrounds in a population-based survey

**DOI:** 10.1186/s13104-021-05775-6

**Published:** 2021-09-10

**Authors:** Husayn Marani

**Affiliations:** 1 Institute of Health Policy, Management and Evaluation, Dalla Lana School of Public Health, University of Toronto, 155 College Street, 4th Floor, Toronto, ON M5T 3M6 Canada; 2 North American Observatory On Health Systems and Policies, University of Toronto, 155 College Street, 4th Floor, Toronto, ON M5T 3M6 Canada

**Keywords:** Caregivers, Minority groups, Sampling studies, Health surveys, COVID-19

## Abstract

**Objective:**

Historically, persons from minority ethnic, religious and linguistic backgrounds have been un- or under-represented in population-based research studies. Emerging scholarship suggests challenges in representative sampling, particularly of minority ethnocultural groups, has been exacerbated by the COVID-19 pandemic. This research note offers additional insights concerning these challenges in the context of a population-based survey of unpaid caregivers conducted in Ontario, Canada, between August and December, 2020, the analysis of which is currently underway.

**Results:**

Beyond limitations intrinsic to study design, including time and budget constraints, the study sample underrepresents unpaid caregivers from minority ethnocultural backgrounds due to differences in conceptions of caregiving across minority cultures, the time-consuming nature of caregiving that disproportionately affects minority groups, and a propensity to avoid research which is rooted in tokenism. These hypotheses are non-exhaustive, speculative and warrant further empirical investigation.

## Introduction

In settings where there is variation across population demographics, aiming to obtain a representative sample in population-based studies is important to establish both internal and external validity [1]. However, emerging health services research suggests that the COVID-19 pandemic may be compromising representative sampling in studies involving participant-facing primary data collection [2], and clinical trials [3]. The abrupt pivot to digital formats to both recruit and collect data from research participants (e.g. through web-based surveys and video interviews) could be widening the digital divide between those with and without access to internet, thereby precluding historically harder-to-reach demographic groups like ethnocultural minorities, from participating in research on the impacts of the pandemic.

One research population this may implicate is unpaid caregivers, who have recently garnered research and policy attention, largely in high-income Western settings, due to an increase in responsibilities caused by pandemic lockdowns and restrictions in the health system [4]. Unpaid caregivers provide medical, emotional, and/or psychological support for a family member, friend or neighbour living with a health condition or limitations in activities of daily living [5]. Historically, unpaid caregivers from minority ethnocultural backgrounds have been underrepresented in population-based studies on caregiving due to the time-consuming nature of caregiving and, consequently, a propensity among researchers to rely on convenience sampling which excludes harder-to-reach populations like minority ethnocultural groups [6, 7].

Although research on the impacts of the pandemic on unpaid caregivers continues to unfold, as of yet there is relative dearth in recent scholarship describing challenges, if any, in representative sampling of unpaid caregivers. In this research note, we aim to substantiate early suggestions that the pandemic is exacerbating challenges in representative sampling of minority ethnocultural groups by sharing insights from our experiences sampling unpaid caregivers to participate in an original population-based survey, the development of which has been described elsewhere [8]. Based on these challenges, we offer three reasons why minority ethnocultural caregivers may be underrepresented in our study and other population-based studies on caregiving conducted over the pandemic. We hope caregiving researchers in applicable settings may find these insights useful should pandemic-constraints continue to shape data collection approaches.

## Main text

### Overview of survey and sampling approach

An anonymous and voluntary web-based survey [8] was conducted between August and December 2020 to understand the financial impacts of caregiving on unpaid caregivers in Ontario, Canada. Ontario has the highest number of unpaid caregivers in Canada at 3.3 million [5], 30% of whom are visible minorities (non-white), and nearly half of whom were born outside Canada or are first-generation Canadians [9]. Next to British Columbia, Ontario is also the most ethnically diverse province in Canada, with three in ten Ontarians identifying as a visible minority, of which the main groups are South Asian (29.6%), Chinese (19.4%), and Black (16.2%) [10]. 30% of all Ontarians speaking a primary language other than English [11].

Due to pandemic constraints that prevented the distribution of paper surveys, the survey was web-based, but participants had the option to complete the survey over the phone with the principal investigator if they preferred. The survey was in English and took, on average, 20 min to complete.

A brief description of this study consisting of a link to the survey as well as a promotional flyer was emailed by the principal investigator to over 100 organizations across Ontario, including arms-length government caregiver agencies, non-profit charities, provincial chapters of health condition-specific societies, respite and adult day programs, provincial community health centres, homecare service provider organizations, and patient and family/caregiver advisory councils at hospitals and regional health networks across Ontario. Word-of-mouth and social media were also used extensively, including Twitter, Facebook, Instagram, LinkedIn, and Reddit.

Although our sampling approach incorporated strategies described in scholarship on engaging ethnically diverse caregivers in research [6, 12], the final study sample underrepresents Ontario’s ethnic, religious and linguistic (“ethnocultural”) diversity. From a raw data sample of 302 participants sampled over a five-month period, only < 19% were non-white, < 8% spoke a language other than English, and < 16% were born outside Canada. Looking at ethnic background specifically, these results are not wholly representative of the proportion of visible minorities across Ontario. This underrepresentation is consistent with other population-based caregiver research utilizing survey methods conducted during the pandemic, in which, despite being conducted in ethnically diverse settings (Canada and United States), less than 30% of respondents are non-white [4, 13–15].

### Limitations in sampling approach intrinsic to study design

Several factors intrinsic to study design including a short recruitment timeline, budget constraints, and a small study team may have precluded participation of unpaid caregivers from minority ethnocultural backgrounds. For example, though lay in language, recruitment material and the survey itself were in English as translation services were not financially feasible. Further, budget constraints prevented us from compensating participants for their time, which has been noted to improve response rate among ethnocultural minorities in electronic surveys during the pandemic [4]. However, participants were invited to enter a raffle to win monetary prizes.

Pandemic-specific constraints also hindered in-person recruitment in areas with a high density of unpaid caregivers, including adult day programs, hospitals, and long-term care facilities. This meant the survey and recruitment materials were fully online, excluding anyone without a broadband internet connection, and potentially deterring ethnocultural minorities who, overall, are more inclined to complete paper-based surveys over web-based surveys [16]. This may explain why participation in an ongoing population survey in the United States dropped among ethnic minorities (Hispanic-Americans) at the start of the pandemic [17].

These limitations are agnostic to caregivers and may explain the underrepresentation of minority ethnocultural groups across a range of population-based surveys. In the following section, we update previous scholarship [6] and offer new ideas to explain why caregivers from minority ethnocultural backgrounds may be underrepresented in population-based surveys, specifically in the context of pandemic research.

### Hypotheses

#### Cultural understanding of caregiving

Recruitment material sought participation from “unpaid caregivers”, a term commonly used in the literature. However, this term may not be ubiquitous across ethnocultural groups. There is growing understanding that, for some Asian, Latin American, and African diasporas, perceptions of activities that may otherwise constitute care may not be perceived as care, but an inherited familial expectation rooted in tradition and family hierarchy [18, 19]. Relatedly, care recipients from minority ethnocultural backgrounds, particularly older adults, may live in multigenerational households. In such cases, care-related activities may be diffused across a greater number of family members from younger generations, who may not consider their activities as caregiving [18, 20].

Hence, individuals who may not self-identify as a caregiver for these reasons may not have come across online recruitment material for this study, or may not have felt eligible to participate. In such cases, determining eligibility cannot be at the sole discretion of the study participant, and researchers should engage in purposive sampling, or exercising judgement about who may be a caregiver based on heuristics techniques, for example, observing interactions between a patient and their possible caregiver at locations such as hospitals [21]. Given pandemic restrictions, caregiving researchers may need to consider alternative approaches to purposive sampling to recruit minority ethnocultural groups.

It is also possible that those who do self-identify as a caregiver living in a multigenerational setting were concerned about privacy issues in participating in this study, whether online on a shared computer or over the phone. This is common in studies with sensitive or personal topics [22]. Moving forward, caregiving researchers should offer participants multiple modes of survey completion [16]; for example, the option to receive a paper survey by mail.

### The nature of caregiving (lack of time)

Unpaid caregiving is a time-consuming activity that sometimes necessitates working caregivers to exit the workforce to provide care full- or part-time. It is also physically and emotionally exhausting [23]. Caregivers may receive respite when their care recipient is at an adult day program, or being cared for by a paid support worker, during which time they may be able to participate in other activities such as research studies. However, factors such as the cost of adult daycare and paid home support, along with challenges in finding culturally and linguistically competent paid support workers, may be barriers to respite for unpaid caregivers from lower income and/or minority ethnocultural backgrounds. This exacerbates already limited time to participate in research.

These barriers to respite have been further exacerbated by the pandemic. Although the boredom and restlessness of increased time spent at home due to pandemic lockdowns may have encouraged research participation [24], this may not be generalizable to unpaid caregivers, particularly those from minority ethnocultural groups who are more likely to be working on the frontlines, and have been disproportionately more affected by COVID-19 [25]. Ruppel (2020) notes that requesting participants to take part in social research during a pandemic is challenging, especially if those participants are directly experiencing the effects of the pandemic [26]. With adult day programs closed and paid support paused, opportunities for respite dwindled [9], and unpaid caregivers in Canada and elsewhere found themselves taking on more responsibilities during the pandemic than before [4, 27].

Given the above challenges, research interest in unpaid caregiving has grown during the pandemic, with caregivers being inundated with research requests at the start of the pandemic. This may have led to research fatigue among caregivers already burned out by their daily activities. This was confirmed by several organizations (specifically those who serve a minority ethnocultural population) we contacted to help with recruitment, who refused on account of having received too many requests to promote such research, and concerns about exploiting their caregiver networks. Caregiving researchers may therefore want to consider shorter web-based surveys that are not cognitively demanding, which may improve response rates among ethnocultural minorities and otherwise [28, 29].

### Propensity to avoid research rooted in tokenism

Conversations with managers of community health organizations that provide health and social services to minority ethnocultural groups often told us they would promote this study, but to be aware that “these communities do not participate in these things”. Further empirical work should be conducted to validate this; however, we know the roots of this sentiment. Historically, persons from minority ethnocultural backgrounds are sometimes, but not always, marginalized or vulnerable, defined as groups that experience exclusion, disadvantage, or struggle economically, socially, politically or health-wise [2]. Those who are advantaged in these regards are more likely to access digital health services, and by extension, participate in online research during the pandemic due to their material wealth, social capital and access to health information (now digital) [30], resulting in research outcomes, including policy changes, that are informed by the insights of an advantaged few. Accordingly, gaps in needs based on users’ experiences are based on a limited pool of users [31]. In the context of minority ethnocultural caregivers, it stands to reason, then, that this elicits feelings of tokenism; they may be unable to discern how their participation in research on how to improve policies and strategies could contribute to meaningful changes that benefit them directly, thus contributing to a propensity to avoid research altogether.

To reduce the risk of tokenism that has historically deterred minority ethnocultural groups from participating in research studies [32], caregiving researchers conducting research during the pandemic should help participants discern how their involvement in research could benefit them [33], enable involvement through ample and equitable opportunities to participate, specifically translating research materials in different languages [34], and compensate participants [35]. Given ongoing pandemic restriction on in-person networking, researchers should also allot ample time to build relationships with gatekeepers (e.g. coordinators at adult day programs) who may be able to facilitate trust between researchers and caregiver groups from minority backgrounds [36].

### Reflections and conclusion

Pragmatically speaking, it is unlikely that any population-based research on unpaid caregiving will be wholly representative of all caregiving experiences, particularly during a pandemic and when constraints around time, budget, and human resources exist. Witt & Schnabel (2020) note that “in time sensitive research […], circumstances may have changed considerably by the time the research proceeds, meaning it may be impossible to collect important empirical materials” [37]. For this reason, the study team felt compelled to cap recruitment at five months given the rapidly changing landscape of the pandemic, and because prolonging data collection meant relying on participant recall, which, given the nature of some study questions, may not have yielded as accurate responses [31].

As pandemic-related challenges in primary data collection will likely last long after the pandemic ends, insights from this research note may help caregiving researchers in applicable settings understand what to consider if aiming for a representative sample, and how to adapt data collection approaches to improve response rates among ethnocultural minorities specifically.

## Limitations

Ideas presented in this note on why unpaid caregivers from minority ethnocultural backgrounds are underrepresented in our study are largely hypothetical and non-exhaustive. Further empirical work should be conducted to validate these hypotheses.

## Data Availability

The datasets used and/or analysed during the current study are available from the corresponding author on reasonable request.

## References

[1] Stanley S (1999). Science, ethnicity, and bias: where have we gone wrong?. Am Psychol.

[2] Hall J, Gaved M, Sargent J (2021). Participatory research approaches in times of Covid-19: a narrative literature review. Int J Qual Methods.

[3] Gill PS, Poduval S, Thakur JS, Iqbal R (2021). COVID-19, community trials, and inclusion. Lancet.

[4] Irani E, Niyomyart A, Hickman RL (2021). Family caregivers’ experiences and changes in caregiving tasks during the COVID-19 pandemic. Clin Nurs Res.

[5] Arriagada P. The experiences and needs of older caregivers in Canada. Statistics Canada. 2020. https://www150.statcan.gc.ca/n1/pub/75-006-x/2020001/article/00007-eng.htm. Accessed 28 Apr 2021.

[6] Amador TK, Travis SS, McAuley WJ, Bernard M, McCutcheon M (2006). Recruitment and retention of ethnically diverse long-term family caregivers for research. J Gerntol Soc.

[7] Pruchno RA, Brill JE, Shands Y, Gordon JR, Genderson MW, Rose M, Cartwright F (2008). Convenience samples and caregiving research: how generalizable are the findings?. Gerontologist.

[8] Marani H, Allin S, Marchildon G. Development of a web-based survey on the financial risks of unpaid caregiving: approach and lessons learned from a Canadian perspective. Home Health Care Serv Q. Forthcoming 2021. 10.1080/01621424.2021.1976344 34581238

[9] Ontario Caregiver Organization. 3rd annual spotlight on ontario's caregivers—COVID-19 edition. 2020. https://ontariocaregiver.ca/wp-content/uploads/2020/12/OCO-Spotlight-report-English-Dec10.pdf. Accessed 25 Apr 2021.

[10] Ministry of Finance. 2016 Census Highlights. 2016. Office of Economics Policy. https://www.fin.gov.on.ca/en/economy/demographics/census/cenhi16-9.pdf. Accessed 29 Jul 2021.

[11] Statistics Canada. Proportion of mother tongue responses for various regions in Canada, 2016 Census. 2019. https://www12.statcan.gc.ca/census-recensement/2016/dp-pd/dv-vd/lang/index-eng.cfm. Accessed 28 Apr 2021

[12] Wasilewski MB, Stinson JN, Webster F, Cameron JI (2019). Using Twitter to recruit participants for health research: an example from a caregiving study. Health Inform J.

[13] Baumbusch J, Bennett-Lamden SR, Lloyd JEV. The Impact of COVID-19 on British Columbia's children with medical complexity and their families. 2020. doi: 10.14288/1.0395118

[14] Cohen SA, Kunicki ZJ, Drohan MM (2021). Exploring changes in caregiver burden and caregiving intensity due to COVID-19. Gerontol Geriatr Med.

[15] Oxfam, Promundo-US, MenCare. Caring under COVID-19: how the pandemic is—and is not—changing unpaid care and domestic work responsibilities in the United States. 2020. https://oxfamilibrary.openrepository.com/bitstream/handle/10546/621084/FINAL%20REPORT.pdf?sequence=1. Accessed 29 Jul 2021.

[16] Hagan TL, Belcher SM, Donovan HS (2017). Mind the mode: differences in paper vs web-based survey modes among women with cancer. J Pain Symptom Manage.

[17] Rothbaum J. How Does the pandemic affect survey response: using administrative data to evaluate nonresponse in the current population survey annual social and economic supplement. The United States Census Bureau; 2020. https://www.census.gov/newsroom/blogs/research-matters/2020/09/pandemic-affect-survey-response.html. Accessed 29 Jul 2021.

[18] Pharr JR, Francis CD, Terry C, Clark MC (2014). Culture, caregiving, and health: exploring the influence of culture on family caregiver experiences. Int Sch Res Not.

[19] Andruske CL, O'Connor D (2020). Family care across diverse cultures: re-envisioning using a transnational lens. J Aging Stud.

[20] D’Amen B, Socci M, Santini S (2021). Intergenerational caring: a systematic literature review on young and young adult caregivers of older people. BMC Geriatr.

[21] Dilworth-Anderson P, Williams IC, Gibson BE (2002). Issues of race, ethnicity, and culture in caregiving research: a 20-year review (1980–2000). Gerontologist.

[22] Masri AA, Masannat M. Data collection in Covid-19 restrictions. In: GAGE. 2020. https://www.gage.odi.org/multimedia/data-collection-in-covid-19-restrictions/. Accessed 28 Apr 2021.

[23] Gérain P, Zech E (2019). Informal caregiver burnout? Development of a theoretical framework to understand the impact of caregiving. Front Psychol.

[24] Lupton D. (2020). Doing fieldwork in a pandemic. https://docs.google.com/document/u/1/d/1clGjGABB2h2qbduTgfqribHmog9B6P0NvMgVuiHZCl8/mobilebasic#heading.h.am6e9k1kpbda. Accessed 26 Apr 2021.

[25] Statistics Canada. Impacts on immigrants and people designated as visible minorities. 2020. https://www150.statcan.gc.ca/n1/pub/11-631-x/2020004/s6-eng.htm. Accessed 28 Apr 2021

[26] Ruppel S. When your lab is the world but the world is closed down—Social science research in times of Covid-19. Elephant in the Lab. 2020. Doi: 10.5281/zenodo.3885618

[27] Béland D, Marier P (2020). COVID-19 and long-term care policy for older people in Canada. J Aging Soc Policy.

[28] Yetter G, Capaccioli K (2010). Differences in responses to Web and paper surveys among school professionals. Behav Res Methods.

[29] Jang M, Vorderstrasse A (2019). Socioeconomic status and racial or ethnic differences in participation: web-based survey. JMIR Res Protoc.

[30] van Deursen AJ (2020). Digital inequality during a pandemic: quantitative study of differences in COVID-19-related internet uses and outcomes among the general population. J Med Internet Res.

[31] Vindrola-Padros C, Chisnall G, Cooper S, Dowrick A, Djellouli N, Symmons SM (2020). Carrying out rapid qualitative research during a pandemic: emerging lessons from COVID-19. Qual Health Res.

[32] Ocloo J, Matthews R (2016). From tokenism to empowerment: progressing patient and public involvement in healthcare improvement. BMJ Qual Saf.

[33] Hahn DL, Hoffmann AE, Felzien M, LeMaster JW, Xu J, Fagnan LJ (2017). Tokenism in patient engagement. Fam Pract.

[34] Premji S, Kosny A, Yanar B, Begum M (2020). Tool for the meaningful consideration of language barriers in qualitative health research. Qual Health Res.

[35] Ejiogu N, Norbeck JH, Mason MA, Cromwell BC, Zonderman AB, Evans MK (2011). Recruitment and retention strategies for minority or poor clinical research participants: lessons from the Healthy Aging in Neighborhoods of Diversity across the Life Span study. Gerontologist.

[36] Forbat L (2003). Concepts and understandings of dementia by ‘gatekeepers’ and minority ethnic ‘service users’. J Health Psychol.

[37] Witt A, Schnabel S. When the “field” is closed: (Field) research as crisis practice in the times of corona. PRIF Blog. 2020. https://www.hsfk.de/publikationen/publikationssuche/publikation/wenn-das-feld-geschlossen-ist-feld-forschung-als-krisenpraxis-in-zeiten-von-corona/. Accessed 28 Apr 2021.

